# Supplementary material for: Dialysis Patients Respond Adequately to Influenza Vaccination Irrespective of Dialysis Modality and Chronic Inflammation
Source: J Clin Med. 2023 Sep 26;12(19):6205. doi: 10.3390/jcm12196205 (PMC10573409; doi:10.3390/jcm12196205)
Supplement: Supplementary file 1 [file jcm-12-06205-s001.zip › jcm-2610945-supplementary.pdf]

RESPONSE H1N1

```
>
> r1<-
glm(ResponseH1N1~Group+Age+Gender+Diabetes+IL1+IL6+Monthsondialysis+HD
access+HDFvolume, family=binomial)
> summary(r1)

Call:
glm(formula = ResponseH1N1 ~ Group + Age + Gender + Diabetes +
    IL1 + IL6 + Monthsondialysis + HDaccess + HDFvolume, family =
binomial)

Deviance Residuals:
    Min       1Q   Median       3Q      Max
-1.6903  -1.1744   0.8127   1.0662   1.3594

Coefficients:
                   Estimate Std. Error z value Pr(>|z|)
(Intercept)      -3.010e+01  1.029e+03  -0.029   0.9767
GroupHD           1.581e+01  7.277e+02   0.022   0.9827
GroupHDF          7.029e-01  7.456e-01   0.943   0.3458
Age              -1.544e-02  6.892e-03  -2.240   0.0251 *
GenderM          -9.824e-02  1.999e-01  -0.492   0.6230
DiabetesY         8.929e-01  2.176e-01   4.103 4.09e-05 ***
IL1              -5.314e-02  1.192e-01  -0.446   0.6557
IL6              -1.206e-02  9.174e-03  -1.315   0.1885
Monthsondialysis  4.758e-03  1.926e-03   2.470   0.0135 *
HDaccessCVC       3.119e+01  1.029e+03   0.030   0.9758
HDaccessFistula   3.093e+01  1.029e+03   0.030   0.9760
HDaccessGraft     3.069e+01  1.029e+03   0.030   0.9762
HDFvolume        -3.090e-02  3.724e-02  -0.830   0.4067
---
Signif. codes:  0 ‘***’ 0.001 ‘**’ 0.01 ‘*’ 0.05 ‘.’ 0.1 ‘ ’ 1

(Dispersion parameter for binomial family taken to be 1)

    Null deviance: 778.26  on 567  degrees of freedom
Residual deviance: 738.85  on 555  degrees of freedom
  (188 observations deleted due to missingness)
AIC: 764.85

Number of Fisher Scoring iterations: 14

> anova(r1,test="Chisq")
Analysis of Deviance Table

Model: binomial, link: logit
```

Response: ResponseH1N1

```
Terms added sequentially (first to last)


                 Df Deviance Resid. Df Resid. Dev  Pr(>Chi)
NULL                               567     778.26
Group             2   1.3661       565     776.90 0.5050816
Age               1   2.0321       564     774.87 0.1540099
Gender            1   0.8107       563     774.05 0.3679156
Diabetes          1  12.5528       562     761.50 0.0003956 ***
IL1               1   1.2537       561     760.25 0.2628397
IL6               1   1.2259       560     759.02 0.2682062
Monthsondialysis  1   5.9940       559     753.03 0.0143545 *
HDaccess          3  13.4909       556     739.54 0.0036868 **
HDFvolume         1   0.6912       555     738.85 0.4057405
---
Signif. codes:  0 ‘***’ 0.001 ‘**’ 0.01 ‘*’ 0.05 ‘.’ 0.1 ‘ ’ 1
>
======================================================================
===================
```

PROTECTION H1N1

```
> p1<-
glm(ProtectionH1N1~Group+Age+Gender+Time+Diabetes+IL1+IL6+Monthsondial
ysis+HDaccess+HDFvolume, family=binomial)
Warning message:
glm.fit: fitted probabilities numerically 0 or 1 occurred
> summary(p1)

Call:
glm(formula = ProtectionH1N1 ~ Group + Age + Gender + Time +
    Diabetes + IL1 + IL6 + Monthsondialysis + HDaccess + HDFvolume,
    family = binomial)

Deviance Residuals:
     Min        1Q    Median        3Q       Max
-2.37324   0.00000   0.00000   0.00006   1.09755

Coefficients:
                   Estimate Std. Error z value Pr(>|z|)
(Intercept)       1.897e+00  6.029e+04   0.000   1.0000
GroupHD           1.994e+01  3.115e+04   0.001   0.9995
GroupHDF         -6.960e+00  4.167e+00  -1.670   0.0949 .
Age              -9.348e-03  1.929e-02  -0.485   0.6280
GenderM           2.576e-01  5.348e-01   0.482   0.6300
Time              1.902e+01  1.450e+03   0.013   0.9895
DiabetesY        -5.359e-01  5.484e-01  -0.977   0.3285
IL1               3.181e+00  3.436e+03   0.001   0.9993
IL6               3.983e-02  3.011e-02   1.323   0.1860
```

```
Monthsondialysis  2.181e-05  4.483e-03   0.005   0.9961
HDaccessCVC      -2.939e-01  6.029e+04   0.000   1.0000
HDaccessFistula   5.648e-01  6.029e+04   0.000   1.0000
HDaccessGraft     6.864e-01  6.029e+04   0.000   1.0000
HDFvolume         3.338e-01  2.242e-01   1.489   0.1365
---
Signif. codes:  0 '***' 0.001 '**' 0.01 '*' 0.05 '.' 0.1 ' ' 1

(Dispersion parameter for binomial family taken to be 1)

    Null deviance: 196.73  on 543  degrees of freedom
Residual deviance: 117.48  on 530  degrees of freedom
  (212 observations deleted due to missingness)
AIC: 145.48

Number of Fisher Scoring iterations: 22

> anova(p1,test="Chisq")
Analysis of Deviance Table

Model: binomial, link: logit

Response: ProtectionH1N1

Terms added sequentially (first to last)


                 Df Deviance Resid. Df Resid. Dev Pr(>Chi)
NULL                              543     196.73
Group             2    1.410      541     195.32  0.49416
Age               1    0.610      540     194.71  0.43465
Gender            1    0.204      539     194.50  0.65178
Time              1   68.431      538     126.07  < 2e-16 ***
Diabetes          1    0.558      537     125.52  0.45494
IL1               1    0.463      536     125.05  0.49625
IL6               1    1.666      535     123.39  0.19685
Monthsondialysis  1    0.006      534     123.38  0.94030
HDaccess          3    2.119      531     121.26  0.54801
HDFvolume         1    3.780      530     117.48  0.05186 .
---
Signif. codes:  0 '***' 0.001 '**' 0.01 '*' 0.05 '.' 0.1 ' ' 1


======================================================================
===========
ResponseH3N2


> r2<-
glm(ResponseH3N2~Group+Age+Gender+Diabetes+IL1+IL6+Monthsondialysis+HD
```

```
access+HDFvolume, family=binomial)
> summary(r2)

Call:
glm(formula = ResponseH3N2 ~ Group + Age + Gender + Diabetes +
    IL1 + IL6 + Monthsondialysis + HDaccess + HDFvolume, family =
binomial)

Deviance Residuals:
    Min       1Q   Median       3Q      Max
-1.4671  -0.8570  -0.6885   1.1544   2.0054

Coefficients:
                   Estimate Std. Error z value Pr(>|z|)
(Intercept)      -8.835e-01  1.697e+03  -0.001 0.999585
GroupHD          -1.678e+01  1.200e+03  -0.014 0.988842
GroupHDF          3.758e+00  1.375e+00   2.733 0.006270 **
Age               7.418e-03  7.636e-03   0.971 0.331326
GenderM          -7.205e-01  2.223e-01  -3.241 0.001192 **
DiabetesY         2.515e-01  2.321e-01   1.083 0.278641
IL1              -2.846e+00  1.237e+02  -0.023 0.981653
IL6               3.553e-02  1.011e-02   3.516 0.000439 ***
Monthsondialysis  1.655e-03  1.718e-03   0.964 0.335267
HDaccessCVC      -4.756e-01  1.697e+03   0.000 0.999776
HDaccessFistula   1.855e-01  1.697e+03   0.000 0.999913
HDaccessGraft    -6.337e-01  1.697e+03   0.000 0.999702
HDFvolume        -2.241e-01  7.273e-02  -3.081 0.002063 **
---
Signif. codes:  0 '***' 0.001 '**' 0.01 '*' 0.05 '.' 0.1 ' ' 1

(Dispersion parameter for binomial family taken to be 1)

    Null deviance: 696.63  on 567  degrees of freedom
Residual deviance: 634.61  on 555  degrees of freedom
  (188 observations deleted due to missingness)
AIC: 660.61

Number of Fisher Scoring iterations: 15

> anova(r2,test="Chisq")
Analysis of Deviance Table

Model: binomial, link: logit

Response: ResponseH3N2

Terms added sequentially (first to last)


                 Df Deviance Resid. Df Resid. Dev  Pr(>Chi)
```

```
NULL                                  567     696.63
Group              2  10.3133         565     686.32 0.0057610 **
Age                1   1.2249         564     685.09 0.2684061
Gender             1   6.4694         563     678.62 0.0109747 *
Diabetes           1   0.0041         562     678.62 0.9491660
IL1                1   5.3598         561     673.26 0.0206064 *
IL6                1  11.7029         560     661.56 0.0006240 ***
Monthsondialysis   1   1.3888         559     660.17 0.2386102
HDaccess           3  11.6260         556     648.54 0.0087805 **
HDFvolume          1  13.9346         555     634.61 0.0001893 ***
---
Signif. codes:  0 ‘***’ 0.001 ‘**’ 0.01 ‘*’ 0.05 ‘.’ 0.1 ‘ ’ 1
>
```

==
================

ProtectionH3N2

```
> p2<-
glm(ProtectionH3N2~Group+Age+Gender+Time+Diabetes+IL1+IL6+Monthsondial
ysis+HDaccess+HDFvolume, family=binomial)
> summary(p2)

Call:
glm(formula = ProtectionH3N2 ~ Group + Age + Gender + Time +
    Diabetes + IL1 + IL6 + Monthsondialysis + HDaccess + HDFvolume,
    family = binomial)

Deviance Residuals:
    Min       1Q   Median       3Q      Max
-3.3553   0.0551   0.1533   0.3027   0.7938

Coefficients:
                   Estimate Std. Error z value Pr(>|z|)
(Intercept)       5.701e+00  5.484e+03   0.001  0.99917
GroupHD           1.562e+01  3.016e+03   0.005  0.99587
GroupHDF         -4.457e+00  3.980e+00  -1.120  0.26270
Age              -4.010e-02  2.226e-02  -1.802  0.07155 .
GenderM          -4.007e-02  5.973e-01  -0.067  0.94651
Time              7.601e-01  2.598e-01   2.926  0.00344 **
DiabetesY         2.618e-01  6.227e-01   0.420  0.67423
IL1               2.317e+00  3.333e+02   0.007  0.99445
IL6              -7.327e-03  2.511e-02  -0.292  0.77044
Monthsondialysis  1.705e-03  5.022e-03   0.340  0.73414
HDaccessCVC      -3.581e-01  5.484e+03   0.000  0.99995
HDaccessFistula  -6.332e-01  5.484e+03   0.000  0.99991
HDaccessGraft     4.361e-01  5.484e+03   0.000  0.99994
HDFvolume         2.094e-01  2.161e-01   0.969  0.33252
---
Signif. codes:  0 ‘***’ 0.001 ‘**’ 0.01 ‘*’ 0.05 ‘.’ 0.1 ‘ ’ 1
```

```
(Dispersion parameter for binomial family taken to be 1)

    Null deviance: 158.11  on 543  degrees of freedom
Residual deviance: 131.23  on 530  degrees of freedom
  (212 observations deleted due to missingness)
AIC: 159.23

Number of Fisher Scoring iterations: 17

> anova(p2,test="Chisq")
Analysis of Deviance Table

Model: binomial, link: logit

Response: ProtectionH3N2

Terms added sequentially (first to last)


                 Df Deviance Resid. Df Resid. Dev  Pr(>Chi)
NULL                               543     158.11
Group             2   0.7198       541     157.39   0.69775
Age               1   4.2721       540     153.12   0.03874 *
Gender            1   0.1402       539     152.97   0.70813
Time              1  17.7107       538     135.26 2.572e-05 ***
Diabetes          1   0.5260       537     134.74   0.46830
IL1               1   0.1785       536     134.56   0.67266
IL6               1   0.0550       535     134.50   0.81466
Monthsondialysis  1   0.1184       534     134.39   0.73075
HDaccess          3   1.7046       531     132.68   0.63590
HDFvolume         1   1.4518       530     131.23   0.22824
---
Signif. codes:  0 ‘***’ 0.001 ‘**’ 0.01 ‘*’ 0.05 ‘.’ 0.1 ‘ ’ 1
>
======================================================================
================
ResponseYamagata
>
> r3<-
glm(ResponseYamagata~Group+Age+Gender+Diabetes+IL1+IL6+Monthsondialysi
s+HDaccess+HDFvolume, family=binomial)
> summary(r3)

Call:
glm(formula = ResponseYamagata ~ Group + Age + Gender + Diabetes +
    IL1 + IL6 + Monthsondialysis + HDaccess + HDFvolume, family =
binomial)

Deviance Residuals:
```

```
    Min       1Q   Median       3Q      Max
-2.0729  -1.2336   0.6961   0.8998   1.3713

Coefficients:
                   Estimate Std. Error z value Pr(>|z|)
(Intercept)      -3.197e+01  1.029e+03  -0.031 0.975214
GroupHD           1.422e+01  7.277e+02   0.020 0.984412
GroupHDF          2.375e-01  8.906e-01   0.267 0.789747
Age              -7.628e-04  7.371e-03  -0.103 0.917572
GenderM           5.297e-01  2.116e-01   2.503 0.012329 *
DiabetesY         1.833e-01  2.291e-01   0.800 0.423597
IL1              -3.109e-01  1.353e-01  -2.298 0.021565 *
IL6               4.800e-02  1.295e-02   3.708 0.000209 ***
Monthsondialysis  2.158e-03  2.056e-03   1.049 0.293977
HDaccessCVC       3.212e+01  1.029e+03   0.031 0.975100
HDaccessFistula   3.153e+01  1.029e+03   0.031 0.975557
HDaccessGraft     3.147e+01  1.029e+03   0.031 0.975609
HDFvolume         2.814e-02  4.495e-02   0.626 0.531344
---
Signif. codes:  0 ‘***’ 0.001 ‘**’ 0.01 ‘*’ 0.05 ‘.’ 0.1 ‘ ’ 1

(Dispersion parameter for binomial family taken to be 1)

    Null deviance: 726.72  on 567  degrees of freedom
Residual deviance: 667.20  on 555  degrees of freedom
  (188 observations deleted due to missingness)
AIC: 693.2

Number of Fisher Scoring iterations: 14

> anova(r3,test="Chisq")
Analysis of Deviance Table

Model: binomial, link: logit

Response: ResponseYamagata

Terms added sequentially (first to last)


                 Df Deviance Resid. Df Resid. Dev  Pr(>Chi)
NULL                               567     726.72
Group             2  12.9466       565     713.77  0.001544 **
Age               1   1.3725       564     712.40  0.241382
Gender            1   3.0761       563     709.33  0.079450 .
Diabetes          1   1.0391       562     708.29  0.308036
IL1               1   1.2519       561     707.03  0.263188
IL6               1  19.4496       560     687.58 1.033e-05 ***
Monthsondialysis  1   0.6668       559     686.92  0.414174
HDaccess          3  19.3089       556     667.61  0.000236 ***
```

```
HDFvolume         1   0.4091       555     667.20  0.522420
---
Signif. codes:  0 ‘***’ 0.001 ‘**’ 0.01 ‘*’ 0.05 ‘.’ 0.1 ‘ ’ 1
>
```

==
========

ProtectionYamagata

```
>
> p3<-
glm(ProtectionYamagata~Group+Age+Gender+Time+Diabetes+IL1+IL6+Monthson
dialysis+HDaccess+HDFvolume, family=binomial)
Warning message:
glm.fit: fitted probabilities numerically 0 or 1 occurred
> summary(p3)

Call:
glm(formula = ProtectionYamagata ~ Group + Age + Gender + Time +
    Diabetes + IL1 + IL6 + Monthsondialysis + HDaccess + HDFvolume,
    family = binomial)

Deviance Residuals:
     Min        1Q    Median        3Q       Max
-2.59784   0.00000   0.00000   0.00007   1.14905

Coefficients:
                   Estimate Std. Error z value Pr(>|z|)
(Intercept)       3.035e+00  6.062e+04   0.000    1.000
GroupHD           2.003e+01  3.162e+04   0.001    0.999
GroupHDF         -3.344e-01  2.495e+00  -0.134    0.893
Age               1.306e-02  2.431e-02   0.537    0.591
GenderM          -1.700e-01  7.735e-01  -0.220    0.826
Time              1.805e+01  1.467e+03   0.012    0.990
DiabetesY         1.153e+00  1.121e+00   1.028    0.304
IL1               3.298e+00  3.481e+03   0.001    0.999
IL6              -3.197e-02  2.669e-02  -1.198    0.231
Monthsondialysis -1.356e-03  5.217e-03  -0.260    0.795
HDaccessCVC      -1.610e+00  6.062e+04   0.000    1.000
HDaccessFistula  -6.456e-01  6.062e+04   0.000    1.000
HDaccessGraft    -4.523e-01  6.062e+04   0.000    1.000
HDFvolume        -5.724e-03  1.229e-01  -0.047    0.963

(Dispersion parameter for binomial family taken to be 1)

    Null deviance: 99.742  on 543  degrees of freedom
Residual deviance: 65.751  on 530  degrees of freedom
  (212 observations deleted due to missingness)
```

```
AIC: 93.751

Number of Fisher Scoring iterations: 22

> anova(p3,test="Chisq")
Analysis of Deviance Table

Model: binomial, link: logit

Response: ProtectionYamagata

Terms added sequentially (first to last)


                  Df Deviance Resid. Df Resid. Dev  Pr(>Chi)
NULL                                543     99.742
Group              2   0.7135       541     99.029    0.7000
Age                1   0.2364       540     98.792    0.6268
Gender             1   0.1276       539     98.665    0.7209
Time               1  27.5418       538     71.123 1.537e-07 ***
Diabetes           1   1.6384       537     69.485    0.2005
IL1                1   0.2169       536     69.268    0.6414
IL6                1   2.3243       535     66.943    0.1274
Monthsondialysis   1   0.0101       534     66.933    0.9198
HDaccess           3   1.1806       531     65.753    0.7577
HDFvolume          1   0.0021       530     65.751    0.9631
---
Signif. codes:  0 ‘***’ 0.001 ‘**’ 0.01 ‘*’ 0.05 ‘.’ 0.1 ‘ ’ 1


======================================================================
======

ResponseVicto1ia
>
> r4<-
glm(ResponseVicto1ia~Group+Age+Gender+Diabetes+IL1+IL6+Monthsondialysi
s+HDaccess+HDFvolume, family=binomial)
> summary(r4)

Call:
glm(formula = ResponseVicto1ia ~ Group + Age + Gender + Diabetes +
    IL1 + IL6 + Monthsondialysis + HDaccess + HDFvolume, family =
binomial)

Deviance Residuals:
    Min       1Q   Median       3Q      Max
-2.2292  -1.2554   0.6478   0.9670   1.3123

Coefficients:
```

```
                   Estimate Std. Error z value Pr(>|z|)
(Intercept)      -2.930e+01  1.029e+03  -0.028  0.97728
GroupHD           1.391e+01  7.277e+02   0.019  0.98475
GroupHDF         -1.356e+00  8.960e-01  -1.513  0.13019
Age              -4.502e-03  7.390e-03  -0.609  0.54240
GenderM           2.067e-02  2.145e-01   0.096  0.92320
DiabetesY         6.519e-01  2.405e-01   2.711  0.00671 **
IL1              -1.836e-01  1.283e-01  -1.431  0.15254
IL6               1.296e-02  1.135e-02   1.142  0.25364
Monthsondialysis -3.830e-03  1.774e-03  -2.159  0.03087 *
HDaccessCVC       3.126e+01  1.029e+03   0.030  0.97577
HDaccessFistula   2.995e+01  1.029e+03   0.029  0.97679
HDaccessGraft     2.982e+01  1.029e+03   0.029  0.97689
HDFvolume         8.660e-02  4.575e-02   1.893  0.05839 .
---
Signif. codes:  0 ‘***’ 0.001 ‘**’ 0.01 ‘*’ 0.05 ‘.’ 0.1 ‘ ’ 1

(Dispersion parameter for binomial family taken to be 1)

    Null deviance: 726.72  on 567  degrees of freedom
Residual deviance: 667.32  on 555  degrees of freedom
  (188 observations deleted due to missingness)
AIC: 693.32

Number of Fisher Scoring iterations: 14

> anova(r4,test="Chisq")
Analysis of Deviance Table

Model: binomial, link: logit

Response: ResponseVicto1ia

Terms added sequentially (first to last)


                 Df Deviance Resid. Df Resid. Dev  Pr(>Chi)
NULL                               567     726.72
Group             2   1.2526       565     725.47 0.5345631
Age               1   0.4932       564     724.97 0.4824906
Gender            1   0.5441       563     724.43 0.4607591
Diabetes          1  12.1634       562     712.27 0.0004874 ***
IL1               1   1.9612       561     710.31 0.1613798
IL6               1   1.4744       560     708.83 0.2246455
Monthsondialysis  1   8.1302       559     700.70 0.0043533 **
HDaccess          3  29.2565       556     671.45 1.978e-06 ***
HDFvolume         1   4.1241       555     667.32 0.0422757 *
---
Signif. codes:  0 ‘***’ 0.001 ‘**’ 0.01 ‘*’ 0.05 ‘.’ 0.1 ‘ ’ 1
>
```

```
======================================================================
================
ProtectionVictoria

>
> p4<-
glm(ProtectionVictoria~Group+Age+Gender+Time+Diabetes+IL1+IL6+Monthson
dialysis+HDaccess+HDFvolume, family=binomial)
Warning message:
glm.fit: fitted probabilities numerically 0 or 1 occurred
> summary(p4)

Call:
glm(formula = ProtectionVictoria ~ Group + Age + Gender + Time +
    Diabetes + IL1 + IL6 + Monthsondialysis + HDaccess + HDFvolume,
    family = binomial)

Deviance Residuals:
     Min        1Q    Median        3Q       Max
-2.71841   0.00000   0.00000   0.00007   0.85620

Coefficients:
                  Estimate Std. Error z value Pr(>|z|)
(Intercept)      4.493e+00  6.061e+04   0.000   0.9999
GroupHD          1.914e+01  3.155e+04   0.001   0.9995
GroupHDF         1.647e+00  2.123e+00   0.776   0.4380
Age              2.253e-03  2.386e-02   0.094   0.9248
GenderM          2.152e-01  6.985e-01   0.308   0.7580
Time             1.822e+01  1.481e+03   0.012   0.9902
DiabetesY        1.707e-01  7.592e-01   0.225   0.8221
IL1              3.653e+00  3.445e+03   0.001   0.9992
IL6             -4.626e-02  2.749e-02  -1.683   0.0924 .
Monthsondialysis  2.681e-03  6.571e-03   0.408   0.6833
HDaccessCVC     -1.164e+00  6.061e+04   0.000   1.0000
HDaccessFistula  -2.516e+00  6.061e+04   0.000   1.0000
HDaccessGraft   -2.202e+00  6.061e+04   0.000   1.0000
HDFvolume       -7.859e-02  9.997e-02  -0.786   0.4318
---
Signif. codes:  0 ‘***’ 0.001 ‘**’ 0.01 ‘*’ 0.05 ‘.’ 0.1 ‘ ’ 1

(Dispersion parameter for binomial family taken to be 1)

    Null deviance: 115.270  on 543  degrees of freedom
Residual deviance:  77.448  on 530  degrees of freedom
  (212 observations deleted due to missingness)
AIC: 105.45

Number of Fisher Scoring iterations: 22
```

```
> anova(p4,test="Chisq")
Analysis of Deviance Table

Model: binomial, link: logit

Response: ProtectionVictoria

Terms added sequentially (first to last)


                 Df Deviance Resid. Df Resid. Dev  Pr(>Chi)    
NULL                               543    115.270              
Group             2    0.273       541    114.997    0.8722    
Age               1    0.062       540    114.935    0.8040    
Gender            1    0.000       539    114.935    0.9830    
Time              1   33.094       538     81.841 8.783e-09 ***
Diabetes          1    0.039       537     81.803    0.8439    
IL1               1    0.386       536     81.417    0.5345    
IL6               1    1.798       535     79.619    0.1799    
Monthsondialysis  1    0.101       534     79.518    0.7510    
HDaccess          3    1.515       531     78.003    0.6787    
HDFvolume         1    0.555       530     77.448    0.4563    
---
Signif. codes:  0 ‘***’ 0.001 ‘**’ 0.01 ‘*’ 0.05 ‘.’ 0.1 ‘ ’ 1
```
